# Association between osteoporosis and the rate of telomere shortening

**DOI:** 10.18632/aging.206034

**Published:** 2024-07-25

**Authors:** Myung-Hoon Han, Hyuk Sung Kwon, Mina Hwang, Hyun-Hee Park, Jee Hyang Jeong, Kyung Won Park, Eun-Joo Kim, Soo Jin Yoon, Bora Yoon, Jae-Won Jang, Jin Yong Hong, Seong Hye Choi, Seong-Ho Koh

**Affiliations:** 1 Department of Neurosurgery, Hanyang University Guri Hospital, Guri 11923, South Korea; 2 Department of Neurology, Hanyang University Guri Hospital, Guri 11923, South Korea; 3 Department of Translational Medicine, Hanyang University Graduate School of Biomedical Science and Engineering, Seoul 04763, South Korea; 4 Department of Neurology, Ewha Womans University College of Medicine, Seoul 07804, South Korea; 5 Department of Neurology, Dong-A Medical Center, Dong-A University College of Medicine, Busan 49201, South Korea; 6 Department of Neurology, Pusan National University Hospital, Pusan National University School of Medicine and Medical Research Institute, Busan 49241, South Korea; 7 Department of Neurology, Eulji University Hospital, Eulji University School of Medicine, Daejeon 35233, South Korea; 8 Department of Neurology, Konyang University College of Medicine, Daejeon 35365, Republic of Korea; 9 Department of Neurology, Kangwon National University School of Medicine, Chuncheon 24341, South Korea; 10 Department of Neurology, Yonsei University Wonju College of Medicine, Wonju 26426, South Korea; 11 Department of Neurology, Inha University College of Medicine, Incheon 22332, South Korea

**Keywords:** leukocyte telomere length, osteoporosis, interleukin-6, aging, prospective cohort

## Abstract

A shorter leukocyte telomere length (LTL) is reported to be associated with age-related diseases, including osteoporosis. Many studies have tried identifying the association between LTL and osteoporosis, although it remains controversial. This study aimed to determine whether osteoporosis is independently associated with LTL shortening in a prospective longitudinal cohort. The KBASE study is an independent multicenter prospective cohort in South Korea, which began in 2014. We compared the LTL values for each participant at baseline and over a 2-year follow-up period. Boxplots were used to demonstrate the differences in the change in LTL over a 2-year follow-up according to osteoporosis. Multivariable linear regression was conducted to identify whether osteoporosis is independently associated with the rate of telomere shortening. A total of 233 subjects (from 55 to 88 years) from the KBASE cohort were finally enrolled in the study. We observed that the LTL decreased by approximately 1.2 kbp over 2 years. While the LTL decreased as age increased, the rate of LTL shortening did not increase with age. Multivariable linear regression analysis indicated that only osteoporosis was independently associated with rapid LTL shortening over 2 years (B, -8.08; p = 0.038). We sought to identify an association between osteoporosis and LTL shortening in an independent prospective cohort. We found that participants with osteoporosis had significantly faster LTL shortening over 2 years than those without osteoporosis. We hope this study will help elucidate the underlying mechanisms in the relationship between LTL and osteoporosis in the future.

## INTRODUCTION

Telomeres are tandem repeat regions with the TTAGGG sequence located at the ends of chromosomes, which undergo attrition with each somatic cell division [1]. The telomere length indicates the replicative history and the replicative potential of human somatic cells [2]. Leukocyte telomere length (LTL) is a widely used biomarker for physiological aging that is affected by older age, psychosocial stress, and medical comorbidities [3]. A shorter LTL is reported to be associated with older age and age-related diseases, such as cognitive impairment, psychiatric disorders, sleep disorders, and osteoporosis [4, 5]. Osteoporosis, one of the most common chronic metabolic diseases, is at increased risk in the elderly, leading to a public health issue in the aging society. There have been many attempts to determine the association between LTL and osteoporosis in the elderly, although it remains controversial [5–8]. However, we believe that since osteoporosis is a chronic systemic disease associated with proinflammatory cytokines, such as IL-6 and tumor necrosis factor (TNF)-alpha, studies with long-term follow-up periods are needed to determine the link between LTL and osteoporosis.

The Korean Brain Aging Study for the Early Diagnosis and Prediction of Alzheimer’s Disease (KBASE) is an independent ongoing prospective longitudinal cohort that started in 2014 [9–11]. The KBASE study was intended to identify risk and prognostic factors associated with dementia in Alzheimer’s disease (AD). Participants in the KBASE cohort were screened for comorbidities at recruitment, including osteoporosis, and underwent clinical and neuropsychological assessments annually and a full KBASE assessment, including neuroimaging and blood laboratory tests, every two years, as described in the previous KBASE cohort study [4, 11]. Subsequently, we thought it would be meaningful to analyze the relationship between LTL and osteoporosis using the longitudinal prospective KBASE cohort. Therefore, the study aimed to determine whether osteoporosis is independently associated with the rate of LTL shortening.

## RESULTS

### Characteristics of study individuals

**Figure 1:**
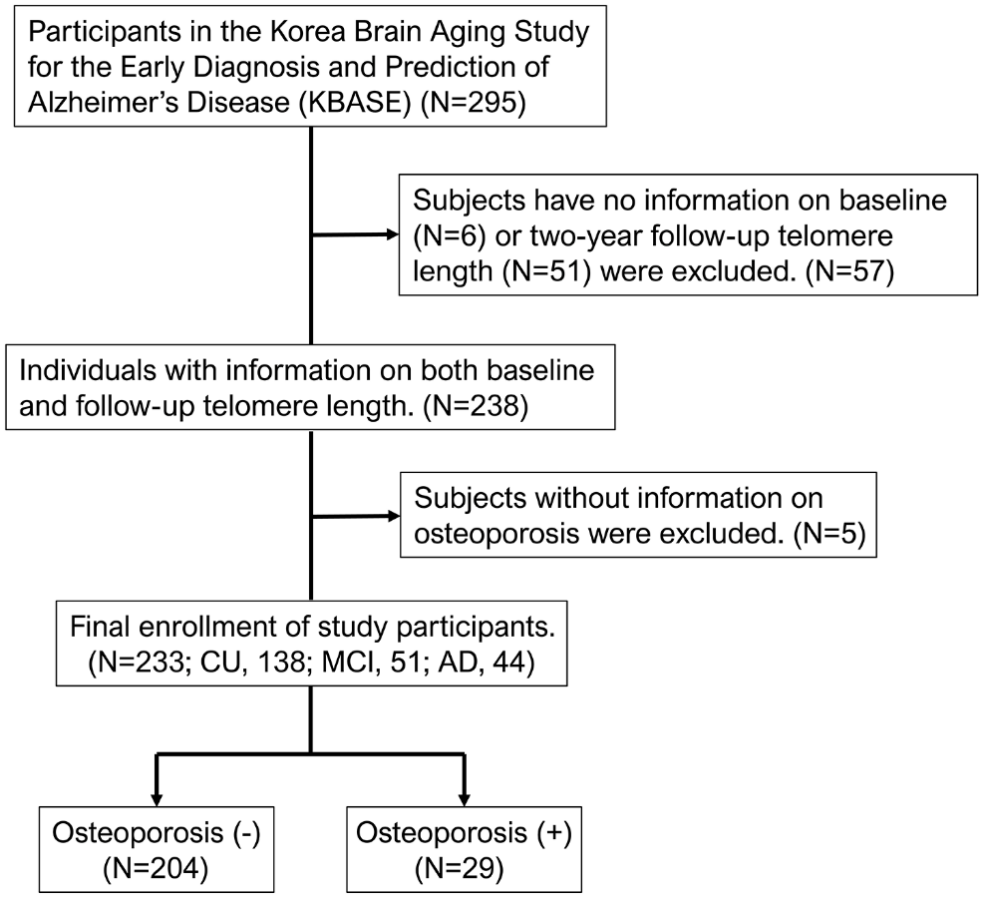
**Flowchart of the analyzed subjects in the KBASE cohort.** KBASE, Korean Brain Aging Study for the Early Diagnosis and Prediction of Alzheimer’s Disease.

A total of 233 subjects (cognitively unimpaired [CU], 138; mild cognitive impairment [MCI], 51; AD, 44) were selected from an independent validation cohort in the KBASE with information on osteoporosis and both LTL at baseline and over a 2-year follow-up (Figure 1). Among them, 29 participants (12.4%) had been diagnosed with osteoporosis at the start of the study (Table 1). Participants in the study ranged in age from 55 to 88, with an average age of 70.2 years, while 60.5% of participants were female. Most of the study participants with osteoporosis were women (93.1%). The mean baseline LTL was 7.9 kbp, the mean LTL in participants without osteoporosis was 7.8 kbp, and the mean LTL in participants with osteoporosis was approximately 8.5 kbp (Table 1). The mean value of LTL at the approximate 2-year follow-up was around 6.7 kbp, the mean LTL without osteoporosis was approximately 6.8 kbp, and the mean LTL with osteoporosis was approximately 6.6 kbp. The mean LTL values at baseline and the approximate 2-year follow-up, categorized by sex, are presented in Supplementary Table 1. At baseline, the mean LTL values were longer in females than in males (p = 0.044), and this difference was statistically significant. However, at the approximate two-year follow-up, there were no significant differences in mean LTL values between males and females. A history of depressive symptoms was significantly higher in participants with osteoporosis. Further descriptive data are shown in Table 1.

**Table 1:** Characteristics of the study subjects.

**Characteristics**	**Osteoporosis (-)**	**Osteoporosis (+)**	**Total**	**p**
Number (%)	204 (87.6)	29 (12.4)	233	
Sex, female, n (%)	114 (55.9)	27 (93.1)	141 (60.5)	<0.001
Age, mean ± SD, y	70.0 ± 8.2	72.0 ± 8.7	70.2 ± 8.3	0.232
LTL at baseline, mean ± SD, kbp	7.8 ± 2.0	8.5 ± 2.5	7.9 ± 2.0	0.080
LTL at baseline, median (IQR), kbp	7.2 (6.7–8.1)	7.8 (6.8–9.3)	7.2 (6.7–8.2)	0.080
LTL at 2-year follow-up, mean ± SD, kbp	6.8 ± 1.0	6.6 ± 0.8	6.7 ± 0.9	0.195
LTL at 2-year follow-up, median (IQR), kbp	6.6 (6.2–7.4)	6.4 (5.8–7.2)	6.6 (6.1–7.4)	0.195
BMI, mean ± SD, kg/m^2^	24.2 ± 2.9	23.8 ± 3.4	24.1 ± 3.0	0.482
Cognitive status, n (%)				0.276
Normal	117 (57.4)	21 (72.4)	138 (59.2)	
MCI	46 (22.5)	5 (17.2)	51 (21.9)	
ADD	41 (20.1)	3 (10.3)	44 (18.9)	
Current or past treatment history for depressive symptoms, n (%)	27 (13.2)	8 (27.6)	35 (15.0)	0.043
Hypertension, n (%)	94 (46.1)	12 (41.4)	106 (45.5)	0.634
Diabetes, n (%)	31 (15.2)	5 (17.2)	36 (15.5)	0.776
Hyperlipidemia, n (%)	73 (35.8)	13 (44.8)	86 (36.9)	0.345
Current use of supplemental vitamin D or osteoporosis medication, n (%)	17 (8.3)	7 (24.1)	24 (10.3)	0.009

ADD, Alzheimer’s disease dementia; BMI, body mass index; IQR, interquartile range; LTL, leukocyte telomere length; MCI, mild cognitive impairment; SD, standard deviation.

### Difference between baseline LTL and LTL over the 2-year follow-up according to age

**Figure 2:**
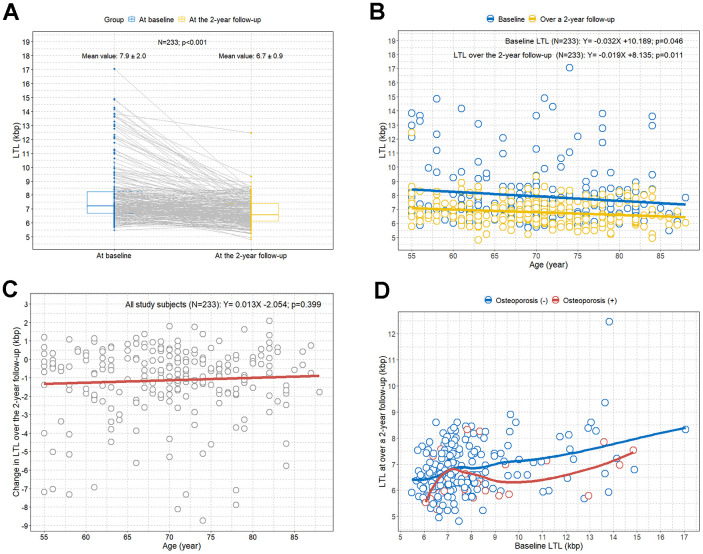
**Comparison of LTL values between baseline and at the 2-year follow-up by age and osteoporosis.** (**A**) Paired comparison of LTL values between at baseline (in blue) and at the 2-year follow-up (in yellow) for each study participant; (**B**) scatterplot with linear regression lines showing the associations between age and LTL; (**C**) scatterplot with linear regression line showing the association between age and change in LTL over a 2-year follow-up; (**D**) scatterplot with LOWESS curves showing the trend between baseline LTL and LTL at the 2-year follow-up according to osteoporosis. LOWESS, locally weighted scatter plot smoothing; LTL, leukocyte telomere length.

The baseline LTL values were compared to the LTL values at the 2-year follow-up in 233 participants. We observed an overall significant decrease in LTL values at the 2-year follow-up compared to the baseline LTL values (Figure 2A). We also observed that both the baseline LTL and LTL at the 2-year follow-up significantly decreased as the age increased (Figure 2B). However, the change in LTL over the 2-year follow-up was not associated with an increase in age (Figure 2C).

### Difference between baseline LTL and LTL at the 2-year follow-up according to osteoporosis

The LOWESS regression analysis revealed an overall trend toward lower LTL values at the 2-year follow-up compared to the baseline LTL in participants with osteoporosis compared to participants without osteoporosis (Figure 2D).

### Rapid LTL shortening in subjects with osteoporosis

**Figure 3:**
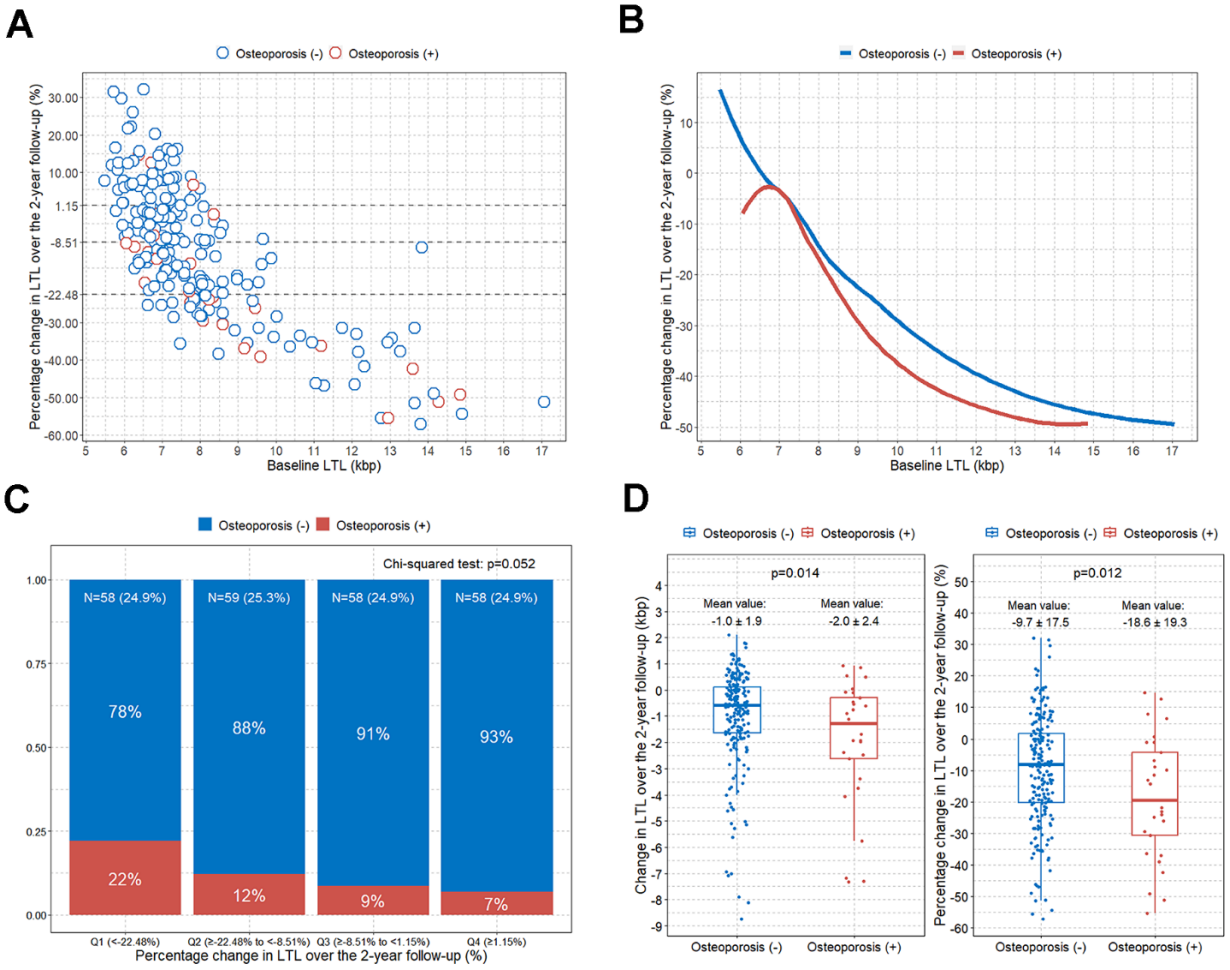
**Comparison of change in LTL over the 2-year follow-up according to osteoporosis.** (**A**) Scatterplot showing the associations between baseline LTL and percentage change in LTL over a 2-year follow-up (the dotted line divides the study participants into quartile groups); (**B**) LOWESS curves showing the trend between baseline LTL and percentage change in LTL over a 2-year follow-up according to osteoporosis; (**C**) bar plot showing the percentage of osteoporosis after dividing the participants into quartile groups according to the LTL percentage change over the 2-year follow-up; (**D**) boxplots with jitters showing comparison of changes in LTL over the 2-year follow-up according to osteoporosis. LOWESS, locally weighted scatter plot smoothing; LTL, leukocyte telomere length.

We calculated the percentage change in LTL at the 2-year follow-up from baseline LTL for all study participants (Figure 3A). When study individuals were stratified by osteoporosis, participants with osteoporosis tended to have a greater percentage reduction in LTL over the 2-year follow-up from the baseline LTL compared to participants without osteoporosis (Figure 3B). When the participants were categorized into quartile groups for the percentage change in LTL over the 2-year follow-up from the baseline LTL, the highest percentage of individuals with osteoporosis was found in the first quartile group (< -22.48%), which showed the largest percentage decrease in LTL (Figure 3C). The percentage of subjects with osteoporosis decreased from the second to the fourth quartile, although it was just below statistical significance (p = 0.052) (Figure 3C). We found a statistically significant greater reduction in LTL over the 2-year follow-up compared to the baseline LTL in the participants with osteoporosis compared to those without osteoporosis (Figure 3D).

### Osteoporosis is independently associated with rapid LTL shortening

The results of the univariable and multivariable linear regression analyses are presented in Table 2. The multivariable linear regression analysis adjusting for all clinical factors showed that only osteoporosis was independently associated with rapid LTL shortening over 2 years (B, -8.08; 95% CI, -15.70 to -0.47; p = 0.038) (Table 2).

**Table 2:** Univariable and multivariable linear regression analyses of percentage changes in telomere length at the 2-year follow-up based on clinical factors.

**Variable**	**Univariable linear regression analysis**		**Multivariable linear regression analysis**	
	**B (95% CI)**	**p**	**B (95% CI)**	**p**
Intercept	N/A		-14.51 (-46.02 to 17.00)	0.365
Male (vs. female)	3.97 (-0.75 to 8.68)	0.099	2.74 (-2.44 to 7.92)	0.298
Age (per 1-year increase)	0.05 (-0.23 to 0.33)	0.716	0.09 (-0.23 to 0.42)	0.577
BMI (per 1 BMI increase)	-0.06 (-0.84 to 0.73)	0.887	-0.03 (-0.88 to 0.83)	0.952
MCI (vs. normal)	-0.65 (-6.25 to 4.96)	0.820	-2.04 (-8.16 to 4.08)	0.513
ADD (vs. normal)	1.05 (-4.87 to 6.97)	0.726	-1.69 (-9.25 to 5.87)	0.660
Treatment history for depressive symptoms	0.81 (-5.680 to 7.29)	0.807	2.19 (-5.10 to 9.49)	0.554
Osteoporosis	-8.88 (-15.80 to -1.95)	0.012	-8.08 (-15.70 to -0.47)	0.038
Hypertension	0.31 (-2.18 to 2.80)	0.808	-0.92 (-5.95 to 4.12)	0.720
Diabetes	-1.81 (-8.21 to 4.60)	0.579	-1.62 (-8.17 to 4.94)	0.627
Hyperlipidemia	-0.98 (-2.70 to 0.75)	0.266	-2.25 (-7.22 to 2.71)	0.372
Current use of supplemental vitamin D or osteoporosis medication	-4.24 (-11.99 to 3.52)	0.283	-2.27 (-10.18 to 5.63)	0.571

ADD, Alzheimer’s disease dementia; BMI, body mass index; CI, confidence interval; MCI, mild cognitive impairment; N/A, not available.

## DISCUSSION

We found that LTL decreased in the study participants aged 55 to 88 years by approximately 1.2 kbp over 2 years. While the LTL decreased with increasing age, the rate of LTL shortening did not increase as age increased. We observed no statistically significant difference in LTL between individuals with and without osteoporosis. However, interestingly, we found that osteoporosis was significantly associated with rapid LTL shortening in an independent prospective cohort. We observed that the LTL was approximately 8% shorter over two years in participants with osteoporosis compared to participants without osteoporosis. To our knowledge, this is the first study to demonstrate faster LTL shortening in patients with osteoporosis by comparing LTL values that were measured twice, approximately 2 years apart, in an independent prospective cohort.

Since telomeres range from approximately 5 to 15 kbp in normal human somatic cells [12], we believe that our results show that when the LTL is measured in independent cohorts at a specific time point, there may not be a significant difference in LTLs between participants with and without osteoporosis. However, when we measured the LTL twice, approximately 2 years apart, in all participants in an independent prospective cohort, a rapid LTL shortening was observed in participants with osteoporosis compared to those without osteoporosis. Since osteoporosis is a systemic disease and the inflammatory response caused by osteoporosis may interact with LTL, it seems important to observe changes in LTL over a period of time in patients with osteoporosis [13]. Since osteoporosis is more prevalent in older individuals, both age and osteoporosis would be confounding factors in LTL values if the LTL were only measured at a single time point. As shown in our study, older age was associated with significantly shorter LTL but not with the rate of LTL shortening. Therefore, unsurprisingly, we think that a longitudinal rather than a cross-sectional study is more appropriate when studying the association between LTL and osteoporosis. This is potentially why controversy exists regarding the association between osteoporosis and LTL shortening [5–7].

It has been reported that inflammation, a pathogenesis of osteoporosis, can accelerate telomere length shortening [14]. In a recently published randomized controlled prospective study, we demonstrated that the systemic inflammatory cytokine IL-6 can influence LTL shortening [15]. Previous studies also reported that the cumulative load of IL-6 can mediate telomere shortening [16–19]. Interestingly, IL-6 is also a cytokine associated with the inflammatory response in osteoporosis [20–22]. Therefore, we hypothesized that increased IL-6 activity in osteoporotic patients may contribute to accelerating LTL shortening. In addition, previous studies have shown that IL-6 levels are elevated in patients with depression [23, 24]. In our study, depressive symptoms were significantly prevalent in participants with osteoporosis (Table 1). Therefore, this evidence could also support that increased IL-6 is associated with LTL shortening. Similar to inflammation, oxidative stress has also been linked to LTL shortening [13]. It has been reported that the rate of LTL shortening correlated with the oxygen concentration [25, 26]. Telomeres are particularly vulnerable to oxidative damage. Telomeric DNA is particularly sensitive to reactive oxygen species (ROS) because its G-rich sequence and oxidized telomere DNA interfere with the telomerase action [13]. In addition, because of the presence of the shelterin complex at telomeres, telomeric damage by ROS may not efficiently activate the DNA damage response proteins [13]. It is known that oxidative stress is also strongly linked to osteoporosis [27, 28]. Therefore, we believe that oxidative stress will accelerate both LTL shortening and bone resorption in humans. Thus, LTL may partly record an individual’s lifetime cumulative burden of inflammation cytokines and oxidative stress, both of which are related to osteoporosis [14].

Our study has some limitations. First, this study only contains information on LTL at the baseline and 2-year follow-up. However, we think more details on additional long-term LTL values would provide further insight into the underlying mechanisms between osteoporosis and LTL. Second, because this study was initially designed to predict Alzheimer’s disease, we did not possess any T-score information. We think the results would have been more accurate and meaningful if T-score information was available at baseline and the 2-year follow-up. Therefore, future studies are needed to validate the association between osteoporosis and LTL further. Third, the inherent study design makes it difficult to establish the causal effects of LTL changes on osteoporosis. Fourth, our study employed a method that only provided the mean LTL, and did not allow for a complete analysis of TL distribution. Recognizing this limitation, a more comprehensive technique such as metaphase Q-FISH could have enhanced our findings [29]. This method offers detailed TL distribution across chromosomes and identifies the percentage of short telomeres per cell, which are crucial for understanding chromosomal instability associated with age-related diseases like osteoporosis. We are planning to utilize this method in our forthcoming research to address these limitations. Lastly, we included only Korean participants with old age and a high proportion of cognitive impairments. Therefore, the current study may limit the generalizability of the results.

## CONCLUSIONS

This study attempted to identify an association between osteoporosis and LTL shortening in an independent prospective cohort. There was no significant difference in LTL between participants with and without osteoporosis. However, we found significantly faster LTL shortening over 2 years in participants with osteoporosis than those without osteoporosis. We hope this study will help elucidate the mechanism underlying the relationship between LTL and osteoporosis in the future.

## MATERIALS AND METHODS

### Study participants

This study analyzed participants from the previously published KBASE study [4, 9–11]. The KBASE study is an independent validation prospective cohort from 2014 that used nine memory clinics across South Korea. All participants in the KBASE study ranged in age from 55 to 90 years, and had reliable informants who could provide the investigators with the requested information. The study was performed in accordance with the International Harmonization Conference guidelines on Good Clinical Practice and the Declaration of Helsinki and approved by the institutional review board of each participating center prior to beginning the study. As previously described [4, 10], the exclusion criteria for this study were as follows: (1) presence of a major psychiatric disorder; (2) serious neurological or medical conditions or comorbidities that could affect cognitive function; (3) contraindications to magnetic resonance imaging (MRI) (e.g., pacemaker, claustrophobia); (4) illiteracy; (5) severe visual or hearing impairments or severe communication or behavioral problems that could make clinical examination or brain scans difficult; (6) taking an investigational drug; (7) pregnant or breastfeeding. We only included study participants with baseline and follow-up LTL measures and information on osteoporosis (Figure 1).

### Clinical assessment

All participants underwent a standardized clinical assessment by an experienced board-certified psychiatrist following the KBASE clinical assessment protocol at the time of recruitment [11]. We also interviewed reliable informants and reviewed medical records to determine whether more accurate information was available. Global and domain-specific Clinical Dementia Rating (CDR) scores and Global Dementia Deterioration Scale (GDS) were administered to all participants to assess dementia severity. Physical and neurological examinations were also conducted by clinicians, as previously described [11]. Current and past medical comorbidities, family history of dementia, medication use, dietary supplements, smoking, alcohol, and coffee consumption were assessed by trained nurses based on structured interviews with participants and their informants. More detailed study methods have been described previously [4, 9–11].

We only defined osteoporosis as a confirmed T-score of -2.5 or less at the femoral neck or lumbar spine on the bone mineral density (BMD) test report at the time of recruitment. Osteoporosis medications included bisphosphonates, calcitonin, estrogen preparations, hormone replacement therapy, selective estrogen-receptor modulators, ipriflavone, and recombinant parathyroid hormone. Treatment history of depressive symptoms was defined as currently receiving treatment or having previously been treated for depressive symptoms. Hypertension was defined as a prescription of antihypertensive medication or measured systolic blood pressure ≥ 140 mm Hg or diastolic blood pressure ≥ 90 mm Hg. Diabetes was defined as a prescription of insulin or oral glucose-lowering medication, high plasma glucose level (≥ 126 mg/dL) after 8 hours of fasting, or high glycated hemoglobin level (≥ 6.5%). Hyperlipidemia was defined as the prescription of lipid-lowering medication or high levels of total cholesterol (≥ 200 mg/dL), low-density lipoprotein cholesterol (≥ 130 mg/dL), triglycerides (≥ 150 mg/dL), and low levels of high-density lipoprotein cholesterol (< 40 mg/dL).

### Leukocyte telomere length assay

Fasting blood samples were obtained from all participants at baseline and approximately 2 years after baseline. As previously described [4, 15], we measured LTL as follows: At baseline, whole blood was collected from individuals, separated into plasma and a buffy coat, and leukocyte DNA was extracted using G-DEXTM IIb RBC lysis buffer and G-DEXTM IIb cell lysis buffer (Intron, MA, USA). Then, the DNA was hydrated, and TLs were measured using a non-radioactive TeloTAGGG TL assay (Roche Boehringer-Mannheim, Grenzach-Wyhlen, Germany), as described by the manufacturer. Briefly, 2–4 μg of DNA was fragmented and separated using agarose gel electrophoresis. Then, the DNA fragments were transferred to a nylon membrane (Millipore, MA, USA) and incubated with digoxigenin (a digoxigenin-labeled probe), which attaches explicitly to telomere repeat sites. Next, the membranes were incubated with secondary antibodies conjugated with alkaline phosphatase. TLs were measured visually using chemiluminescence and an image analyzer (ImageQuant LAS 4000, GE Healthcare, Little Chalfont, UK). TLs were determined by comparison to molecular weight standards. All LTLs were analyzed by two investigators who were blinded to the information of each participant. The intraclass correlation coefficient between the two evaluators was 0.905, indicating excellent reliability [4].

### Statistical methods

Chi-squared and Student’s t-tests were applied for the discrete and continuous variables, respectively, to determine the differences between the non-osteoporotic and osteoporotic groups.

The paired comparison plot was used to show differences in LTL between the baseline and the 2-year follow-up for each study subject. We performed linear regression analysis to evaluate associations between age and LTL and the change in LTL in study individuals over a 2-year follow-up. In addition, to graphically represent the association between the baseline and follow-up LTL values, we used a scatterplot with lines determined by locally weighted scatterplot smoothing (LOWESS). The rationale and detailed methods for using LOWESS were previously described elsewhere [30]. Boxplots with jitters were used to visualize differences in LTL changes over a 2-year follow-up according to osteoporosis in the study cohort.

Multivariable linear regression was also performed to determine whether osteoporosis is independently associated with the rate of telomere shortening. Sex, age (continuous variable), BMI (continuous variable), MCI, AD, history of depressive symptoms, osteoporosis, hypertension, diabetes, hyperlipidemia, and current use of supplemental vitamin D or osteoporosis medication were entered into the multivariable model.

A p-value < 0.05 was considered statistically significant. All statistical analyses were performed using R software version 4.2.2 and SPSS for Windows version 24.0 (IBM, IL, USA).

### Data availability statement

The data that support the findings of this study are available from the corresponding author upon reasonable request.

## Supplementary Material

Supplementary Table 1
