# Supplementary material for: Association between osteoporosis and the rate of telomere shortening
Source: Aging (Albany NY). 2024 Jul 25;16(14):11151–61. doi: 10.18632/aging.206034 (PMC11315396; doi:10.18632/aging.206034)
Supplement: Supplementary Table 1 [file aging-16-206034-s001.pdf]

SUPPLEMENTARY TABLE

Supplementary Table 1. Characteristics of the study subjects according to sex.

Characteristics	Male	Female	Total	p
Number (%)	92 (39.5)	141 (60.5)	233	
Age, mean ± SD, y	71.0 ± 7.5	69.7 ± 8.7	70.2 ± 8.3	0.242
LTL at baseline, mean ± SD, kbp	7.6 ± 1.7	8.1 ± 2.2	7.9 ± 2.0	0.044
LTL at baseline, median (IQR), kbp	7.1 (6.6–8.0)	7.3 (6.8–8.6)	7.2 (6.7–8.2)	0.044
LTL at 2-year follow-up, mean ± SD, kbp	6.7 ± 0.8	6.8 ± 1.0	6.7 ± 0.9	0.380
LTL at 2-year follow-up, median (IQR), kbp	6.6 (6.2–7.4)	6.6 (6.0–7.4)	6.6 (6.1–7.4)	0.380

IQR, interquartile range; LTL, leukocyte telomere length; SD, standard deviation.
